# Biological Activity of Natural and Synthetic Compounds

**DOI:** 10.3390/molecules27123652

**Published:** 2022-06-07

**Authors:** Carlotta Granchi

**Affiliations:** Department of Pharmacy, University of Pisa, 56126 Pisa, Italy; carlotta.granchi@unipi.it

A drug discovery program starts when a disease or clinical condition has no suitable drugs. In response, the pharmaceutical industry and academic research groups can follow different processes aimed at identifying new molecules with drug-like properties that efficiently modulate the desired biological target. These new chemical entities can be isolated from natural sources or obtained by chemical synthesis, as demonstrated by the articles and review papers reported in this Special Issue.

The natural compound lesbicoumestan, isolated from the flowering plant *Lespedeza bicolor*, showed a promising antileukemia potential by inducing the degradation of mucosa-associated lymphoid tissue lymphoma translocation protein 1 (MALT1), thus leading to the inhibition of the NF-κB signalling pathway, a well-known negative regulatory factor of apoptosis. Molecular docking studies confirmed the binding of lesbicoumestan to the active site of MALT1. Moreover, lesbicoumestan showed apoptotic effects on human leukemia cells by several mechanisms: it increased mitochondrial reactive oxygen species, activated caspase-dependent pathways and decreased the mRNA expression level of antioxidant genes. All these effects contributed to the anti-proliferative activity on Jurkat cells, as also confirmed by three-dimensional Jurkat spheroid models [1]. Diarylheptanoids oregonin, hirsutenone and hirsutanonol, isolated from the deciduous tree *Alnus sibirica*, exerted anti-proliferative activities on androgen-dependent (LNCaP) and androgen-independent (PC-3) prostate cancer cell lines. Among the three compounds, hirsutenone proved to be the most promising potential therapeutic agent against prostate cancer, since it exhibited the highest cytotoxic activity, a significant NF-κB inhibitory ability and it was a potent apoptosis-inducer [2]. In addition to plants, fungi were also shown to be an important source of bioactive compounds, as in the case of terretonin N and butyrolactone I, which are metabolites isolated from the thermophilic fungus *Aspergillus terreus* TM8. Both compounds were cytotoxic on prostate adenocarcinoma (PC-3) and ovary adenocarcinoma (SKOV3) human cell lines by promoting apoptosis [3].

Sferrazza et al. exhaustively reviewed the properties of the herbal plant *Hovenia dulcis Thunberg* (Rhamnaceae family) from a phytochemical, pharmacological, nutritional and toxicological point of view, also giving a hint to the regulatory aspects for the use of *H. dulcis* extract in Europe. *H. dulcis* is considered an herbal remedy in traditional Chinese medicine thanks to its many pharmacological properties (i.e., antidiabetic, anticancer, antioxidant, anti-inflammatory, hepatoprotective and anti-hangover activities), which are caused by several secondary metabolites produced by different parts of the plant, such as dammarane-type triterpene saponins, flavonoids, dihydroflavonols, flavonols, organic acids, polysaccharides and alkaloids [4].

Considering the research field of antibacterial agents, Terreni et al. uncovered the alarming problem of antibiotic resistance and the emergence of multidrug-resistant bacterial strains. A full description of the bacterial and molecular mechanisms of antibiotic inactivation introduced the specific sections about the most innovative molecules active against multidrug-resistant organisms. Finally, the poorly explored strategy of using nanoparticles delivery systems for antibiotics was discussed [5].

An innovative therapeutic strategy was presented by Mostafa et al. who identified *Halomonas meridiana*, a marine bacterium isolated from the Red Sea, as an efficient L-glutaminase producer, which can be considered as a potential anti-colorectal cancer agent. L-glutaminase (which hydrolyzes L-glutamine to L-glutamic acid and ammonia) is an L-glutamine-depleting enzyme, and it exerts its action by depriving cancer cells of this essential nutrient, thus blocking their growth. The purified enzyme produced by *Halomonas meridiana* showed a significant cytotoxic activity in human colorectal adenocarcinoma cells (LS174T and HCT116) [6].

Chemical entities endowed with therapeutic utility can be naturally produced by human body, as in the case of propionic acid, a short-chain fatty acid produced through bacterial fermentation in the gastrointestinal tract. It was shown that propionic acid suppressed cervical cancer cell (HeLa) viability by triggering apoptosis and autophagy [7].

In some cases, a natural compound can be a source of inspiration for the design of synthetic analogues, as in the case of the well-known curcumin isolated from the rhizomes of *Curcuma longa*, and endowed with many useful pharmacological activities but neglected as therapeutic agent due to its low bioavailability. Starting from curcumin’s structure, the synthetic curcuminoid analogue (2E,6E)-2,6-bis(2,3-dimethoxybenzylidine) cyclohexanone (DMCH) was evaluated on colon cancer cell lines HT29 and SW620 for what concerns cytotoxicity, apoptosis induction and the activation of apoptosis-related proteins. Both cell lines showed a reduced growth after DMCH treatment, and the inhibition of proliferation was provoked by apoptosis induction, with a more pronounced effect in the highly metastatic SW620 cells. Interestingly, DMCH was more effective in inducing cell death than the parent compound curcumin, thus confirming the efficacy of this natural product-derived compound [8]. Blažíčková et al. focused their research on a natural compound extracted from the herb *Thymus vulgaris*, thymol, whose use was greatly limited by its hydrophobic properties. Therefore, more hydrophilic derivatives were synthesized or purchased with the aim of improving cell permeability with respect to the parent compound. This strategy led to the identification of acetic acid thymol ester as a promising cytotoxic agent on colorectal cancer cells (HT-29 and HCT-116) [9].

Chemical synthesis was a powerful strategy to obtain new molecules active on a biological target. In this context, chiral neonicotinoids were synthesized and evaluated with the aim of discovering new effective agents against *Xyleborus affinis* beetles. In this study, it was evident that the *R* absolute configuration determined a greater insecticidal property of the compounds, when compared to the opposite enantiomer *S*, thus highlighting that chirality plays a major role in determining biological activity [10]. Modern computational approaches are often successfully used in the drug discovery process, as in the case of Stefanucci et al., who performed a virtual screening study on a large library of compounds, followed by lead optimization and molecular dynamics simulations, to identify tripeptides targeting kappa opioid receptor (KOR). The synthesized hit compounds displayed a good antinociceptive effect in vivo, thus supporting the reliability of the computational protocol [11]. Considering the widely reported biological activities of pyrrolo[1,2-a]quinoline derivatives, a novel series of synthetic compounds possessing this scaffold were screened in vitro on *Candida albicans*, and molecular modeling studies were performed against *C. albicans* pathogenic proteins. Moreover, the exploration of the possible off-targets and ADME parameters were determined by in silico studies, revealing good drug-like properties for the newly identified antifungal molecules [12].

Many bioactive compounds, as well as marketed drugs, possess nitrogen-based heterocyclic structures. In detail, imidazole/fused imidazole rings are the common scaffold of many anticancer agents, as reviewed by Sharma et al. [13], probably due to the features of imidazole heterocycle that are a high polarity and the ability to participate in hydrogen bonds, thus facilitating the interaction with biological targets. Moreover, kinase inhibitors reported in the literature are often characterized by different heterocyclic cores, and, interestingly, most of them found applications in the treatment of neuroblastoma, both at the preclinical and clinical levels [14].

A combined approach merging the isolation of natural compounds and synthesis of new derivatives can be fruitfully exploited to discover new potential therapeutic agents. A bio-assay-guided isolation on the fungus *Albatrellus confluens* against *Caenorhabditis elegans* performed by Dube et al. identified grifolin and neogrifolin as responsible for the anthelmintic activity. Starting from the structures of these two natural compounds, new synthetic analogues were prepared to vary the original chemical structures. Both natural and synthetic derivatives were assayed on a panel of parasites, and some newly synthesized compounds displayed promising antischistosomal activity [15].
